# Supplementary material for: A multiscale view of the Phanerozoic fossil record reveals the three major biotic transitions
Source: Commun Biol. 2021 Mar 8;4:309. doi: 10.1038/s42003-021-01805-y (PMC7977041; doi:10.1038/s42003-021-01805-y)
Supplement: Supplementary file 2 — Supplementary Information [file 42003_2021_1805_MOESM2_ESM.pdf]

# Supplementary Information for

## A multiscale view of the Phanerozoic fossil record reveals the three major biotic transitions

**Alexis Rojas, Joaquin Calatayud, Michal Kowalewski, Magnus Neuman, and Martin Rosvall**

**Alexis Rojas.**
**E-mail: alexis.rojas-briceno@umu.se**

### This PDF file includes:

Supplementary Text
Supplementary Figures 1 to 3
Supplementary Table 1
Supplementary Legends for Supplementary Data 1 to 4
Supplementary References

### Other supplementary materials for this manuscript include the following:

Supplementary Data 1 to 4

## Supplementary Text

**Robustness of the modular structures to the selected relax rate (*r*).** The flow-based nature of map equation multilayer framework enable interlayer coupling based on the intralayer information of the assembled multilayer network. The only methodological decision at hand is to decide the relax rate ($r$) to employ in the clustering analysis. Following previous studies on complex networks (1), we used a relax rate $r = 0.25$ for the reference solution. This relax rate $r$ value is large enough to enable interlayer (temporal) interdependencies but small enough to preserve intralayer (geographic) information. However, a wide range of relax rates ($r$) gives solutions that are similar to the reference solution and results are particularly robust in the domain $r$ 0.20 (Supplementary Figure 3).

**Variability in Infomap solutions.** The inherent variability in Infomap solutions was explored using methods outlined in (2) and was found to be small, meaning that the solution landscape is peaked. By generating 100 partitions we found two clusters of partitions using a distance threshold of 0.01, with codelengths 6.253293 and 6.253988 for the best (smallest codelength) partition in each partition cluster respectively. The distance between these partitions is 0.041065, as measured by the weighted average of the minimum Jaccard distance (details in (2)). This means that the two partition clusters are very similar.

**Bootstrapped networks.** We generated bootstrap replicates of the assembled network by resamping taxon occurrences at a given geographic cell using a truncated Poisson distribution with mean equal to the number of taxon occurrences. The truncated distribution has all probability mass between one and the total number of collections in the grid cell, thus avoiding false negatives. We obtained a resampled link weight for each link in the assembled network by dividing the sampled number by the total number of recorded collections in the grid cell. At the end of the Supplementary Information we have included MATLAB code showing how we created bootstrap networks (Supplementary Code).

## Supplementary Legends

### Supplementary Data 1 (Aggregated_Filtered_Data.txt)

Underlying paleontological data in standard text format. The file includes the genus-level occurrence data of the Phanerozoic benthic marine faunas downloaded from the Paleobiology Database (PaleoDB) and aggregated into spatial grid cells.

### Supplementary Data 2 (Multilayer_Network.net)

Network of Phanerozoic benthic marine faunas in multilayer network format. This standard file specifies nodes and links in two different sections. The first section includes the node indexes and names. The second section describes the intralayer link structure; each row includes layer index, source node index, target node index, and link weight. Interlayer links are derived from the intralayer link structure by relaxing the layer constraints on those links with probability $r = 0.25$.

### Supplementary Data 3 (Reference_Solution.tree)

Reference solution in plain text format. This standard file contains the best hierarchical partition of the attempts. Each row begins with the multilevel module assignments of a node in a colon-separated format and ordered from coarse (supermodules) to fine level. Modules within each hierarchical level are sorted by the total amount of flow they contain – their steady state population of random walkers (23). The decimal number is the amount of flow in each node. The last integer corresponds to the index of the node in the multilayer network file (Supplementary Data 1).

### Supplementary Dataset 4 (Robustness_results_Level-3)

Robustness results of the multilayer network analysis of the fossil record of Phanerozoic benthic marine faunas: Third hierarchical level (Level-3).

**Supplementary Table 1.** Robustness results of the multilayer network analysis of the fossil record of Phanerozoic benthic marine faunas: First (Level-1) and second (Level-2) hierarchical levels. Module robustness is expressed as the probability of retrieving a module with a Jaccard similarity index $P_{0.5}$ and $P_{0.7}$.

| Infomap ID | Name | Abrev. | $P_{05}$ | $P_{07}$ |
|---|---|---|---|---|
| 1 | Cenozoic | Cz | 1,00 | 1,00 |
| 1:1 | Neogene | Ng | 1,00 | 0,99 |
| 1:2 | Paleogene | Pg | 1,00 | 0,99 |
| 1:3 | Quaternary | Q | 1,00 | 0,99 |
| 1:4 | Cretaceous 3 | Cr3 | 1,00 | 1,00 |
| 1:5 | Cretaceous 2 | Cr2 | 1,00 | 1,00 |
| 2 | Paleozoic | Pz | 1,00 | 0,98 |
| 2:1 | Permian | P | 1,00 | 0,99 |
| 2:2 | Devonian | D | 1,00 | 0,97 |
| 2:3 | Ordovician | O | 1,00 | 0,99 |
| 2:4 | Silurian | S | 1,00 | 0,94 |
| 2:5 | Carboniferous | C | 1,00 | 0,99 |
| 3 | Mesozoic | Mz | 1,00 | 0,25 |
| 3:1 | Late Jurassic | J2 | 1,00 | 0,98 |
| 3:2 | Triassic | Tr | 1,00 | 1,00 |
| 3:3 | Early Jurassic | J1 | 1,00 | 0,99 |
| 3:4 | Cretaceous 1 | Cr1 | 1,00 | 1,00 |
| 3:5 | — | — | 0,10 | 0,10 |
| 4 | Cambrian | Cm | 1,00 | 1,00 |
| 4:1 | — | — | 1,00 | 1,00 |
| 4:2 | — | — | 0,85 | 0,58 |
| 4:3 | — | — | 0,99 | 0,69 |
| 4:4 | — | — | 0,96 | 0,20 |
| 4:5 | — | — | 0,99 | 0,96 |
| 4:6 | — | — | 0,52 | 0,36 |
| 4:7 | — | — | 0,95 | 0,63 |
| 4:8 | — | — | 0,93 | 0,84 |
| 4:9 | — | — | 0,61 | 0,41 |

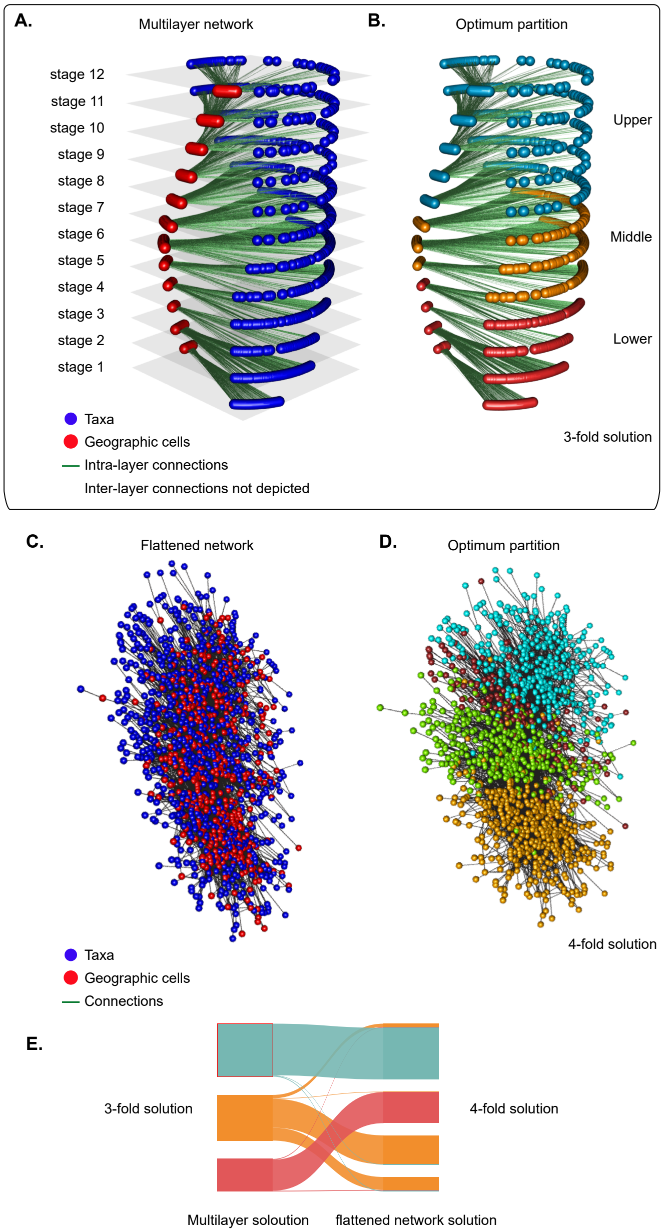


**Supplementary Figure 1. A** multilayer network visualization of fossil occurrence data and its single-layer flattened representation. A. Multilayer network at the stage-level resolution. **B.** Optimum network partition comprising three consecutive modules at the first hierarchical level. In this representation, grid cells are physical nodes restricted to a single layer. One state node in a specific layer represents a grid cell. Taxa are physical nodes that can occur in several layers. One state node per layer represents a taxon. In this visualization, taxa and grid cells are stacked. Each physical node occupies a particular location across all layers, which highlights that taxa gradually replace each other. To avoid clutter, we exclude inter-layer links between state nodes representing grid cells and taxa. **C.** Single-layer flattened representation of the multilayer network. **D.** Optimum network partition comprising four modules at the first hierarchical level. In this network, physical nodes represent taxa and grid cells independent of the stage(s) where they occur. **E.** Alluvial diagram comparing optimum solutions derived from applying Infomap on the multilayer network and its flattened representation. By simplifying this network of networks derived from stage-level resolution data (Supplementary Figure 1A) into an aggregated single-layer network, inevitably we change the trajectory of an entity following the connections of the original network and obscure the macroevolutionary pattern. Visualizations created using the platform MuxViz (58).

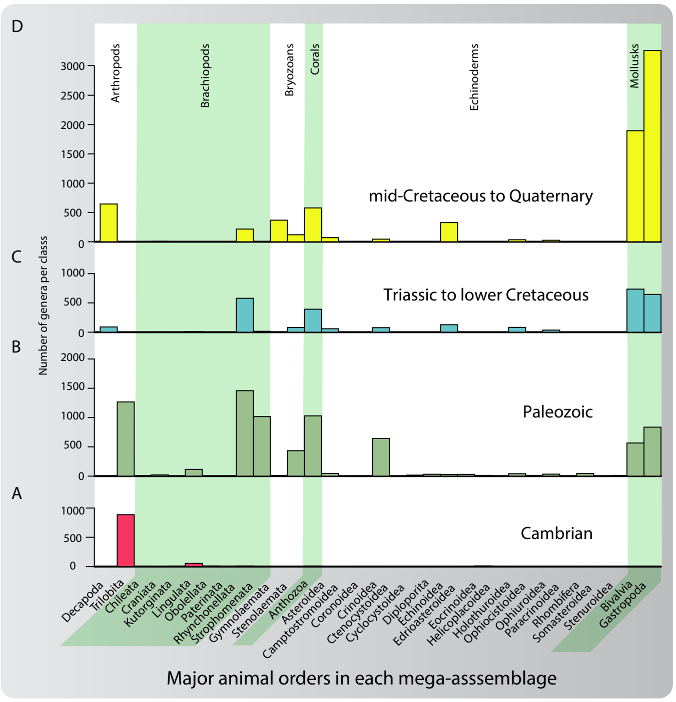


**Supplementary Figure 2.** Class-level composition of the four Phanerozoic marine mega-assemblages. **A.** Cambrian mega-assemblage (Cm). **B.** Paleozoic mega-assemblage (Pz). **C.** Triassic to lower Cretaceous mega-assemblage (Tr-lKr). **D.** Mid-Cretaceous to Quaternary mega-assemblage (mKr-Q). The classes of marine invertebrates that contribute the most to the Cambrian, Paleozoic, and combined post-Paleozoic mega-assemblages delimited here match those from the Three Great Evolutionary Faunas. The Cambrian mega-assemblage comprises trilobites (88%) and lingulates (5%); the Paleozoic mega-assemblage comprises rhynchonellids (19%), trilobites (16%), anthozoans (13%), strophomenids (13%), gastropods (11%), crinoids (8%), bivalves (7%), and stenolaemate bryozoans (6%); the Triassic to lower Cretaceous mega-assemblage comprises bivalves (25%), gastropods (22%), rhynchonellids (20%), and anthozoans (13%); and the mid-Cretaceous to Quaternary mega-assemblage comprises gastropods (43%), bivalves (25%), decapods (8%), and anthozoans (8%).

S3

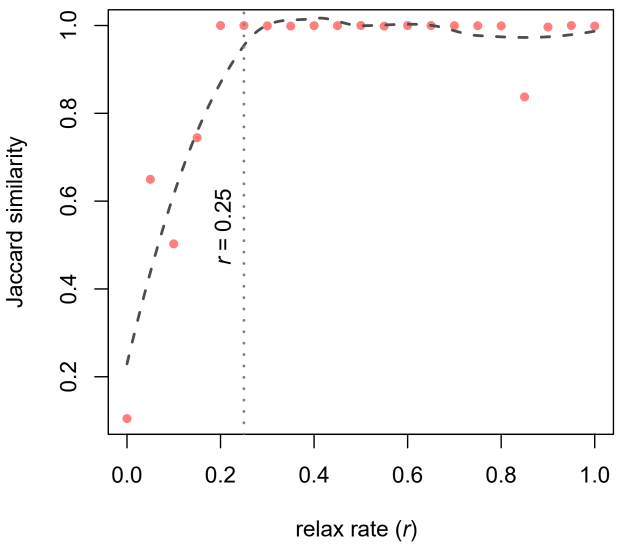


**Supplementary Figure 3.** Robustness of the modular structures to the selected relax rate (*r*). The similarity of the reference solution (*r* = 0.25) to bootstrapped solutions obtained using a wide range of relax rates shows that modular structures representing Phanerozoic marine mega-assemblages in the assembled network are highly robust to variations in *r*.

### Supplementary References

**58.** M De Domenico, MA Porter, A Arenas, MuxViz: a tool for multilayer analysis and visualization of networks. *J. Complex Networks* **3**, 159–176 (2015).

**Supplementary Algorithm**

Matlab code used to generate bootstrap replicates of the assembled network. The procedure is described in the Robustness analysis section.

```
%% create bootstrapped networks
% genusData - genus, grid cells, no. of collections in grid cell ...
    where genus is found, no. of total collections in grid cell
nLinks = size(genusData, 1);
gridCells = unique(genusData(:,2));
nGridCells = length(gridCells);
nNodes = nGridCells + length(unique(genusData(:,3))); % also ...
    genus nodes
genusDataResampled = genusData;
genusWeighted = zeros(size(genusData, 1), 4);
genusWeighted(:, 1:3) = genusData(:, 1:3); % copy layer and ...
    genus numbers
nBootstraps = 100;
for bootstrapN = 1:nBootstraps
    digenuslay(bootstrapN)
    % truncated bootstrap
    for i = 1:nGridCells
        genusThisIndex = find(genusData(:, 2) == gridCells(i)); ...
            % this grid cell
        for j = 1:length(genusThisIndex)
            if genusData(genusThisIndex(j), 4) == ...
                genusData(genusThisIndex(j), 5) % if genus found ...
                in all collections, don't resample
                genusDataResampled(genusThisIndex(j), 4) = ...
                    genusData(genusThisIndex(j), 4);
            else
                pd = makedist('Poisson','lambda', ...
                    genusData(genusThisIndex(j), 4));
                pd_trunc = truncate(pd, 1, ...
                    genusData(genusThisIndex(j), 5)); % truncate ...
                    between 1 and max value
                genusDataResampled(genusThisIndex(j), 4) = ...
                    random(pd_trunc); % number of collections ...
                    where this genus is found
            end
        end
        genusWeighted(genusThisIndex, 4) = ...
            genusDataResampled(genusThisIndex, ...
            4)./genusDataResampled(genusThisIndex, 5); % normalize
    end
    bsFile = ...
        strcat('bsnets/Multilayer_Network_bootstrapped',num2str(bootstrapN),'.net');
    copyfile('Multilayer_Network.net',bsFile);%'Multilayer_Network_bootstrapped.net')
    dlmwrite(bsFile,genusWeighted,'-append','delimiter',' ')
end
```
