# Supplementary material for: A multiscale view of the Phanerozoic fossil record reveals the three major biotic transitions
Source: Commun Biol. 2021 Mar 8;4:309. doi: 10.1038/s42003-021-01805-y (PMC7977041; doi:10.1038/s42003-021-01805-y)
Supplement: Supplementary file 3 — Description of Additional Supplementary Files [file 42003_2021_1805_MOESM3_ESM.pdf]

Description of Additional Supplementary Files

File Name: Supplementary Data 1

Description: Underlying paleontological data in standard text format. The file includes the genus-level occurrence data of the Phanerozoic benthic marine faunas downloaded from the Paleobiology Database (PaleoDB) and aggregated into spatial grid cells.

File Name: Supplementary Data 2

Description: Network of Phanerozoic benthic marine faunas in multilayer network format. This standard file specifies nodes and links in two different sections. The first section includes the node indexes and names. The second section describes the intralayer link structure; each row includes layer index, source node index, target node index, and link weight. Interlayer links are derived from the intralayer link structure by relaxing the layer constraints on those links with probability r = 0.25. SI.

File Name: Supplementary Data 3

Description: Reference solution in plain text format. This standard file contains the best hierarchical partition of the attempts. Each row begins with the multilevel module assignments of a node in a colon-separated format and ordered from coarse (supermodules) to fine level. Modules within each hierarchical level are sorted by the total amount of flow they contain –their steady state population of random walkers (23). The decimal number is the amount of flow in each node. The last integer corresponds to the index of the node in the multilayer network file (Data S1).

File Name: Supplementary Data 4

Description: Robustness results of the multilayer network analysis of the fossil record of Phanerozoic benthic marine faunas: Third hierarchical level (Level-3)
